# Goji Berry in the Diet of the Rabbit Buck: Effects on Semen Quality, Oxidative Status and Histological Features of the Reproductive Tract

**DOI:** 10.3390/antiox12111959

**Published:** 2023-11-02

**Authors:** Gabriele Brecchia, Gerald Muça, Albana Munga, Laura Menchetti, Livio Galosi, Giacomo Rossi, Olimpia Barbato, Grazia Pastorelli, Stella Agradi, Valentina Serra, Majlind Sulçe, Enkeleda Ozuni, Luigj Turmalaj, Marta Castrica, Maria Rachele Ceccarini, Federica Riva, Bernard Fioretti, Alda Quattrone, Maria Laura Marongiu, Giulio Curone

**Affiliations:** 1 Department of Veterinary Medicine and Animal Sciences, University of Milan, Via dell’Università 6, 26900 Lodi, Italy; gabriele.brecchia@unimi.it (G.B.); grazia.pastorelli@unimi.it (G.P.); stella.agradi@unimi.it (S.A.); valentina.serra@unimi.it (V.S.); federica.riva@unimi.it (F.R.); alda.quattrone@unimi.it (A.Q.); giulio.curone@unimi.it (G.C.); 2 Faculty of Veterinary Medicine, Agricultural University of Tirana, Kodër Kamëz, 1029 Tirana, Albania; gmuca@ubt.edu.al (G.M.); amunga@ubt.edu.al (A.M.); msulce@ubt.edu.al (M.S.); enkeleda.ozuni@ubt.edu.al (E.O.); lturmalaj@ubt.edu.al (L.T.); 3 School of Biosciences and Veterinary Medicine, University of Camerino, Via Circonvallazione 93/95, 62024 Matelica, Italy; giacomo.rossi@unicam.it; 4 Department of Veterinary Medicine, University of Perugia, Via San Costanzo 4, 06126 Perugia, Italy; olimpia.barbato@unipg.it; 5 Department of Comparative Biomedicine and Food Science, University of Padova, Agripolis, Viale dell’Università 16, 35020 Legnaro, Italy; marta.castrica@unipd.it; 6 Department of Pharmaceutical Science, University of Perugia, 06123 Perugia, Italy; mariarachele.ceccarini@unipg.it; 7 Department of Chemistry, Biology and Biotechnologies, University of Perugia, Via dell’Elce di Sotto 8, 06123 Perugia, Italy; bernard.fioretti@unipg.it; 8 Department of Veterinary Medicine, University of Sassari, Via Vienna 2, 07100 Sassari, Italy; marongiu@uniss.it

**Keywords:** *Lycium barbarum*, rabbit buck, semen quality, antioxidant capacity, seminal plasma, reproductive organs, testis

## Abstract

Goji berry (GB) shows beneficial effects on human health, although its effects on the male rabbit have been little investigated. This study examines the impact of GB dietary supplementation on the semen traits, antioxidant capacity of seminal plasma, and histological features of the reproductive tract of rabbit buck. Eighteen rabbits were distributed into two dietary groups: one receiving a commercial feed (Control), and the other a feed supplemented with 1% of GB (Goji). After a nutritional adaptation period of 60 days, the animals were subjected to semen collection every 15 days. The semen traits, libido, antioxidant, and inflammatory parameters were collected and analyzed. The rabbits were sacrificed after 60 days, and tissues of the genital tract were analyzed. Compared to the Control group, the Goji group showed higher spermatozoa concentration, motility, and vitality (*p* < 0.05), as well as fewer abnormal spermatozoa and a higher libido (*p* < 0.1). Histological features such as functional activity and hyperplasia were improved by GB and correlated with some semen traits (*p* < 0.05). Conversely, antioxidant and anti-inflammatory parameters were unaffected by the diet. These findings suggest that GB acts on the tissues of the reproductive tract positively influencing semen quality, although further studies are needed to understand the effect on oxidative stress.

## 1. Introduction

Goji berry (GB) *(Lycium barbarum*) has been used for thousands of years as food and medicine in Asian countries, especially in China [1]. In recent decades, this fruit has become popular also in Western countries where it is marketed as a functional food [2]. The berry is rich in protein, carbohydrates, dietary fiber, macro- and micronutrients while containing low levels of fat [3]. GB exhibits a wide range of health promoting effects for humans, including anti-aging [4], anticancer [5], immune enhancement [6], protective for various organs [7], and therapeutic against different diseases [8]. Moreover, GB shows significant antioxidant potential, preventing the production of reactive oxygen species (ROS) and, as a consequence, DNA, lipids, and protein damage, by metal chelation or interactions with other antioxidant molecules [9]. The antioxidant properties of GB can be attributed to the presence of several biologically active compounds including polysaccharides, flavonoids, quercetin in particular, and carotenoids such as lycopene [10,11,12]. 

These health-protective benefits of GB can be exerted in many contexts. Indeed, ROS are generated under various physiochemical conditions, and their balance with antioxidants is always necessary. In particular, the imbalance between ROS and antioxidants is also implicated in the alteration of reproductive functions, and it is linked to male infertility, a health issue that significantly increased in recent decades [13]. Several risk factors can induce oxidative stress and, thus, have a role in the reduction of fertility, such as lifestyle [14], work environment [15,16], dietary behaviors with an imbalance of the n-6/n-3 fatty acids ratio [17,18,19], stress [20], chronic diseases [21], and environmental pollutants [22,23]. 

The spermatozoa and testis of mammalian species are rich in long-chain polyunsaturated fatty acids that are essential for several physiological processes, such as spermatozoa maturation, motility, and acrosome reaction [24,25]. However, the high content of polyunsaturated fatty acids in the spermatozoa membrane and mitochondria makes it particularly vulnerable to free radical attack that initiates lipid peroxidation and results in ROS generation. ROSs intervene in various physiological processes such as spermatozoa maturation, motility, chemotaxis, capacitation, hyperactivation, and acrosome reaction. Nonetheless, when produced in excessive concentration, ROS can lead to several types of oxidative damage, resulting in the loss of functionality and, ultimately, the death of spermatozoa [26]. Thus, the oxidation of polyunsaturated fatty acids in spermatozoa can cause deleterious effects on semen quality (spermatozoa damage, compromised DNA integrity, and increased apoptosis) and functions (altered spermatozoa motility, morphological abnormalities, and compromised sperm–oocyte interactions), ultimately resulting in reduced fertility [14,27,28]. 

New innovative strategies can be employed to mitigate the risk of oxidative stress, thereby improving reproductive functions in both humans and animals. There is evidence that fruits and vegetables within the Mediterranean diet [29] and substances with antioxidant potential, such as polyphenols, flavonoids, carotenoids, l-carnitine, selenium, coenzyme Q10, ubiquinol, resveratrol, and vitamin C, can improve seminal plasma oxidative stress and semen quality [30,31,32]. Recently, studies have been performed in various animal models using diets supplemented with nutraceutical substances such as flaxseed, algae [19], bovine colostrum [33], and GB [34,35,36,37]. Regarding GB, the effect of its derived products has been investigated in vitro [38,39], while dietary supplements with GB have been primarily studied in rodent models [40,41,42,43,44]. However, the in vitro studies are not completely satisfactory because the tissue lacks innervation and vascularization, and the effect on spermatogenesis is not considered. On the other hand, standardization of diets in humans is difficult and, therefore, the utilization of experimental models for in vivo nutritional studies is essential. The rabbit is an excellent animal model to study different physiological functions, including the impact of nutritional supplementations and aspects related to reproduction [45,46,47,48,49]. It has already been employed in experimental trials to evaluate the effects of dietary supplementation with GB on reproductive performance, energy balance, and meat quality [50,51,52,53]. Conversely, only a few studies have evaluated the effect of GB on the male reproductive system of different species [54,55,56,57,58,59] and, in particular, the research activity in the rabbit buck is very limited. However, the rabbit buck is a good model to study mature spermatozoa because the semen can be easily and continuously collected using an artificial vagina without the sacrifice of the animal. In addition, the rabbit is also a valid model for studying semen alterations caused by infection and/or inflammation [60]. 

We hypothesized that GB supplementation may influence the male reproductive tract and have an impact on the oxidative stress of semen and its quality. Accordingly, for the first time, this study investigated the effect of GB dietary supplementation on the reproductive parameters of the rabbit buck using a multidisciplinary approach that included different analytical techniques. Semen quality parameters, total antioxidant capacity, the activity of antioxidant enzymes, and inflammatory cytokines in the seminal plasma, in addition to the histological features of the reproductive tract, were investigated.

## 2. Materials and Methods

### 2.1. Animals and Experimental Design

The experiment was performed at the Agricultural University of Tirana, Faculty of Veterinary Medicine, Albania. The experimental protocol was conducted with the permission of the Ministry of Agriculture and Rural Development, National Authority of Veterinary and Plants Protection (prot. 824/2021) of Albania. All efforts were made to minimize animal suffering and to use only the number of animals necessary to produce consistent results. Moreover, the responsible veterinarian for the farm assessed the health and welfare of the rabbits on a daily basis.

Thirty New Zealand white rabbit bucks, aged 7 months and weighing between 3.4 and 4.4 kg, were individually housed under controlled environmental conditions with a lighting schedule of 16 h of light and 8 h of darkness.

The rabbit bucks were randomly divided into two experimental groups (n = 15/group) according to their diets: Control group received a commercial feed, while the Goji group was fed a commercial feed supplemented with 1% of GB. The rabbits were provided 150 g/d of feed and had water ad libitum. The composition of the diets supplied to the rabbits is described in Table 1 and has been previously used in other studies [52,53,61]. 

**Figure 1:**
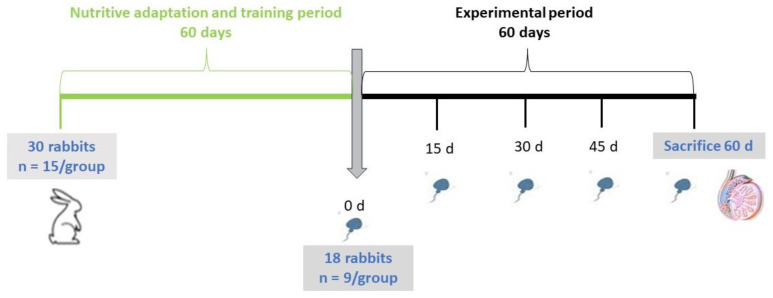
Design of the experimental trial. The green line shows the adaptation and training period (i.e., the animals were fed different diets and trained for semen collection); the black line shows the experimental period (i.e., semen samples were collected every 15 days, and tissues of the reproductive tract were collected after slaughter).

Figure 1 schematizes the experimental design. Thirty animals were divided into two groups (n = 15) and subjected to a nutritional adaptation period of 60 days during which the bucks were also trained for semen collection using an artificial vagina. At the end of this period, 18 males skilled in semen collection were randomly selected from the two groups (n = 9 for each group). During a subsequent experimental period of 60 days (a full spermatogenic cycle), semen samples were collected every 15 days to evaluate several traits (i.e., volume, color, concentration, motility, live spermatozoa, and anomalies), as well as libido. Body weight (BW) measurements were taken before the beginning of the nutritional adaptation and training period, at the beginning of the experimental period (0 d), and at the time of slaughter (60 d).

### 2.2. Sampling of Semen and Reproductive Organs

Throughout the experimental period, a total of five semen samples per rabbit buck were collected at specific time intervals (days 0, 15, 30, 45, and 60; Figure 1). The semen samples were collected using an artificial vagina with a maintained temperature range of 38–40 °C, regularly monitored and adjusted after each ejaculation. Following collection, the samples were divided into two aliquots: one was used to perform the spermatozoa quality evaluation (spermiogram) and the second was centrifuged at 3500 rpm for 15 minutes (Centrifuge 5418 R, Hamburg, Germany). Seminal plasma was recovered, placed in Eppendorf tubes (Eppendorf, Hamburg, Germany), and stored at −20 °C until the determination of the oxidative capacity and inflammatory status. Libido was calculated as “reaction time” by measuring the time (in seconds) between the introduction of the “teaser” doe into the male’s cage and the first ejaculation [63].

At the end of the trial, the rabbits were sacrificed. The testes, epididymis, vesicle glands, prostate, and bulbourethral glands were accurately collected. The samples were fixed in 10% buffered formalin for histological evaluation.

### 2.3. Seminal Quality Assessment

After collection, the semen was immediately subjected to analyses to determine the following seminal traits:

- Volume (mL): determined by graduated tubes;
- Color: defined as milky (normal), cream, yellowish, or transparent;
- Presence of gel (yes or no);
- Concentration (10^6^ spermatozoa/mL): measured with the direct cell count method using a Burker chamber with a 40× objective after dilution of the semen 1:100;
- Motility (%): evaluated by placing two drops of fresh semen on a warm microscope slide and covering it with a glass cover slip. The percentage of motility was calculated after the evaluation of at least 10 microscopic fields with 100× magnification and 10 microscopic fields with 400× magnification;
- Live spermatozoa (%): determined using an eosin–nigrosin blue staining mixture and counting 200 cells. Specifically, 10 µL of undiluted semen was combined with 10 µL of eosin–nigrosin in a 1 mL Eppendorf tube (Eppendorf, Hamburg, Germany), gently mixed, and placed on a microscopic slide for evaluation. Cells that excluded the eosin stain, appearing white, were classified as live cells, while those with compromised or damaged membranes, colored with eosin and appearing pink, were considered dead;
- Abnormal spermatozoa (%): determined using an eosin–nigrosin blue staining mixture and counting 200 cells. Abnormalities were evaluated in all cases, focusing on the abnormal head and tail defects.

### 2.4. Oxidative and Inflammatory Status of Seminal Plasma

The total antioxidant capacity to ROS (mM Trolox equivalents) was analyzed in the seminal plasma samples using the commercial kit Antioxidant Assay Kit (catalog no. 709001; Cayman Chemical, Ann Arbor, MI, USA). The principle of the assay is the ability of antioxidants present within a sample to inhibit the oxidation of ABTS^®^ (2,2’-azino-di-3-ethylbenzthiazoline sulphonate) to ABTS^®+^ by metmyoglobin. The antioxidants cause suppression of absorbance at 750 nm or 405 nm proportionally to their concentration; this capacity to prevent ABTS^®^ oxidation is compared to that of standard Trolox, a water-soluble tocopherol analogue, and is expressed as mM Trolox equivalent (mM TE eq.). The range of detection for this test was 0.068–0.495 mM TE eq.

The activity of three antioxidant enzymes in rabbit seminal plasma was determined with an enzyme-linked immunosorbent assay (ELISA) using commercial kits following the manufacturer’s instructions: catalase (CAT; Catalase Assay Kit, catalog no. 707002; Cayman Chemical, Ann Arbor, MI, USA), superoxide dismutase (SOD; Superoxide Dismutase Assay Kit, catalog no. 706002; Cayman Chemical, Ann Arbor, MI, USA), and glutathione peroxidase (GPx; catalog no. 703102; Cayman Chemical, Ann Arbor, MI, USA).

The Catalase Assay Kit is based on the reaction of the enzyme CAT with methanol in the presence of an optimal H_2_O_2_ concentration. This reaction produces formaldehyde which is measured colorimetrically with the chromogen Purpald that forms a bicyclic heterocycle with aldehydes, changing from colorless to purple upon oxidation. 

The Superoxide Dismutase Assay Kit measures all three types of SOD (Cu/Zn, mn, and FeSOD) using a tetrazolium salt for the detection of superoxide radicals generated by xanthine oxidase and hypoxanthine. One unit of SOD is defined as the amount of enzyme needed to exhibit 50% dismutation of the superoxide radical. The range of detection for this test was 0.005–0.050 U/mL.

The Glutathione Peroxidase Assay Kit measures the activity of GPx in the sample indirectly by a coupled reaction with glutathione reductase; the reduction of hydroperoxide by GPx produces oxidized glutathione which is recycled to its reduced state by glutathione reductase and NADPH (subsequently oxidized to NADP^+^). One unit of GPx is defined as the amount of enzyme that oxidizes 1.0 nmol of NADPH to NADP+ per 1 min at 25 °C. 

The concentration of interleukin-1β (IL-1β) was detected using a commercial ELISA test (Rabbit IL-1β ELISA Kit; FineTest, Wuhan Fine Biotech Co., Ltd., Wuhan, China) based on a sandwich binding in which capture antibody was pre-adsorbed on the surface of microtiter wells. The colorimetric reaction, catalyzed by streptavidin-conjugated horseradish peroxidase (HRP), produced a yellow product, which was proportional to the amount of target molecules present in the sample. The range of detection for this test was 15.625–1000 pg/mL.

The absorbance values of all assays were measured using a spectrophotometer (Epoch Biotek, Agilent Technologies Inc., Santa Clara, CA, USA) at the wavelengths established in each manufacturer’s protocol.

### 2.5. Histological Analysis

The histological examination was performed on the male genital tracts and specifically on the testis, epididymis, seminal vesicles, prostate, and bulbourethral glands. Samples from one rabbit belonging to the Goji group were not evaluated because of technical problems. Briefly, after the rabbits’ sacrifice, the abovementioned organs were extracted and fixed in a 10% neutral buffered formalin solution. The tissue samples were then processed for histological examination with paraffin embedding. Three micrometer-thick sections from paraffin blocks of each sample were stained by hematoxylin and eosin technique. Stained preparations were investigated with an optical microscope (Leica DM2500, Wetzlar, Germany) using 20× magnification. The microscopic evaluation of each sample was conducted by a pathologist who was totally unaware of the division of the two experimental groups, having no information regarding whether the observed sample belonged to the group of treated rabbits or the control group. For each organ sampled, the histological score took into consideration the following parameters: phlogosis, degeneration, functional activity, hyperplasia, and necrosis. To assess the severity of each parameter, a scoring system ranging from 0 to 3 points was employed. This scale allowed for the evaluation of the progressive severity of each parameter, as indicated below. 

Phlogosis: 0 = 5–19; 1 = 20–49; 2 = 50–100; 3 = >100 leucocytes per high-power field (40×). 

Degeneration: 0 = absence of cellular damage; 1 = presence of cytoplasmic vacuolization in some epithelial or neuronal cells; 2 = diffuse cellular vacuolization; 3 = diffuse vacuolization and necrosis/with areas of epithelial cells detachment (groups of cells with microerosive status)/neuronal cells loss. 

Functional activity: 

(A) For prostate, bulbourethral glands, and spermatic vesicles: 0 = glands (tubule-alveolar) with alveoli lined by cuboidal epithelium, collecting ducts lined by cuboidal or columnar epithelium without any trace of secretum; 1 = alveoli lined with cuboidal epithelium, collecting ducts lined with cuboidal epithelium, with a trace of secretum inside some of the alveoli; 2 = alveoli lined with columnar epithelium, hyperplastic epithelium, collecting ducts lined with columnar epithelium with the presence of secretum; 3 = alveoli lined with hyperplastic columnar epithelium, collecting ducts lined with columnar epithelium, with abundant secretum and *corpora amylacea*. 

(B) For testes and epididymis: 0 = Developing spermatogenic cells are only seen in several tubules, and the cell density is increased in the intratubular space. In other tubules, Sertoli cells line the base of the tubules, and spermatogonia are rarely seen in the tubules. The epididymis appears to be characterized as having over 30% empty tubules. The tubules with spermatozoa appear to be characterized by cells that are not very concentrated and localized in the central part of the tubules. 1 = Developing spermatogenic cells are seen in more than 50% of tubules, and the cell density is increased in the intratubular space. The epididymis appears to be characterized by having approximately 20% empty tubules. The tubules with spermatozoa appear to be characterized by cells that are not very concentrated and localized in the central part of the tubules. 2 = Developing spermatogenic cells are seen in more than 60% of tubules, and the cell density is increased in the intratubular space. The epididymis appears to be characterized as having approximately 10% empty tubules. The tubules with spermatozoa appear to be characterized by very concentrated cells and homogeneously localized within the tubules. 3 = Developing spermatogenic cells are seen in all spermatogenic tubules of the *rete testis*, and the cell density is very abundant in the intratubular space. The epididymis appears to be characterized by tubules that are all full, and the spermatozoa form a very concentrated ball of sperm in the tubule, which appears totally full. The Leydig cells that are located between the seminiferous tubules are evident and gathered in small groups, leaving only a few irregular polyhedral cells in rows along with the blood vessels.

Hyperplasia: 

(A) For prostate, bulbourethral glands, and spermatic vesicles: 0 = normal aspect of the epithelium, cells in monolayer; 1 = epithelial cells from cuboidal to columnar, with a trace of secretum; 2 = focal area of stratified columnar epithelium and a diffuse presence of secretum; 3 = diffuse increase in the number of columnar and stratified glandular epithelial cells, with an abundance of secretum. The proliferating epithelium may form papillary structures with supporting stroma and an extension into the glandular lumen. In both scores 2, and 3, there is nuclear crowding, occasional mitotic figures, piling up, and slight basophilia of the hyperplastic epithelium. 

(B) For testes and epididymis: 0 = Normal Sertoli cells line the base of the tubules, and spermatogonia are rarely seen in the tubules. The epididymal tubules show a normal cuboidal or flattened epithelium. 1 = Focal areas of Sertoli hyperplastic cells line the base of the tubules, and spermatogonia are normally seen in the tubules. The epididymis tubules show a normal cuboidal epithelium. 2 = Diffuse hyperplastic Sertoli cells line the base of the tubules, and spermatogonia and spermatids are normally seen in the tubules. The epididymis tubules show a normal cuboidal epithelium with areas of columnar epithelium. 3 = Diffuse hyperplastic Sertoli cells line the base of the tubules, and spermatogonia and spermatids are normally seen in the tubules. The epididymis tubules show a diffuse columnar hyperplastic epithelium.

Necrosis: 0 = absence of necrosis; 1 = few and small areas of necrosis; 2 = diffuse but well-defined areas of tissue necrosis; 3 = diffuse and coalescing areas of tissue necrosis. 

### 2.6. Statistical Analyses

The normality was verified with the Kolmogorov–Smirnov test, while diagnostic graphs were used to identify outliers. Root square transformation was used for CAT, GPx, IL-1β, abnormal spermatozoa, and reaction time; arcsine transformation was used for motility, while the complement to 100 was calculated for live cells percentage. The raw data are reported in the tables as the means and standard errors (SEs). Two values for the CAT, 1 for GPx, 1 for IL-1β, and 2 values for the reaction time were considered outliers and eliminated. Data were then analyzed using generalized estimating equations assuming an exchangeable working correlation matrix and evaluating the effect of group (2 levels: Control and Goji), time (5 levels), and their interaction. The time was included as a repeated measure and rabbits as subjects. Sidak correction was used for multiple comparisons. The normal distribution with identity link was used for the parameters related to the oxidative status, volume, concentration, motile cells, and abnormal spermatozoa; gamma with log link was used for reaction time, whereas negative binomial distribution with log link was used to analyze live cells. Color differences of the semen were analyzed at each time using Fisher’s exact tests.

The associations between the group and histological scores were evaluated with the eta coefficient. The eta is a measure of nominal-by-interval association that ranges from 0 to 1, with 0 indicating no association among the variables, and values close to 1 indicating a high degree of association [64]. In particular, as a measure of the effect size, the eta was interpreted as a small association if it was <0.3, medium if 0.3 ≤ eta < 0.5, and large if eta was ≥ 0.5 [65]. Moreover, eta^2^ (the percent of variance in the dependent variable explained by the group variable [64] was calculated for large effect sizes. The results of the Fisher’s exact and z tests (that compare the column proportions) are also reported.

Finally, the correlations between seminal parameters, TAC, antioxidant enzymes, interleukin, and histological scores were evaluated using Spearman’s rho coefficient (ρ). The correlation was considered poor if ρ < |0.3|, medium if |0.3|≤ ρ < |0.5|, and large if ρ ≥|0.5| [66]. Histological scores were correlated with the mean values of the other parameters. Necrosis and phlogosis were not included because they were constant.

Statistical analyses were performed with SPSS Statistics version 25 (IBM, SPSS Inc., Chicago, IL, USA). We defined *p* < 0.05 as significant and 0.05 ≤ *p* < 0.1 as a trend.

## 3. Results

### 3.1. Clinical Evaluation and Body Weight

The clinical status and welfare of rabbit bucks, daily evaluated by the responsible veterinarian, were good during the entire trial period (120 days: nutritional adaptation and experimental period) in both groups. The rabbit’s body weights increased over time (*p* < 0.001), and at slaughter they were higher in the Goji group than in the Control (4.3 ± 0.1 kg and 4.6 ± 0.1 kg for Control and Goji groups, respectively; *p* = 0.007). We assumed that the palatability of the feed supplemented with GB was the same as the Control feed, given that rabbits from both groups consumed the entire daily administered feed. 

### 3.2. Semen Quality Assessment

The semen volume was not influenced by either group or time, while a significant group and/or time effect was found for the other parameters (Table 2). Pairwise comparisons showed no differences between groups within each time point. However, the Goji group showed higher marginal means for concentration, motility, and live spermatozoa, whereas the percentage of abnormal spermatozoa was lower than the Control group (for all: *p* < 0.05). Abnormal spermatozoa, regardless of the group, also increased over time (*p* < 0.001). Finally, the reaction time progressively reduced over time (*p* < 0.001) and tended to be the lowest in the Goji group (*p* < 0.1), indicating an improvement in libido.

Regarding the color, no differences between the groups were found at any time point (Figure S1).

### 3.3. Antioxidant and Inflammatory Parameters in Seminal Plasma

The values for TAC, antioxidant enzymes, and IL-1β at different time points are reported in Table 3. No significant differences due to the nutritional treatment were found for any analyzed parameter. The interaction group × time was significant for TAC, CAT, and SOD (*p* < 0.01). The TAC values for the Control group increased at the last time point (*p* < 0.05), while in the Goji group, the increase was not significant; however, pairwise comparisons did not show differences between groups at any time point. Similarly, CAT and SOD showed a significant interaction effect but, perhaps due to the high variability of the values, the pairwise comparisons did not show significant differences between the groups at any time point. 

### 3.4. Histological Analysis

**Figure 2:**
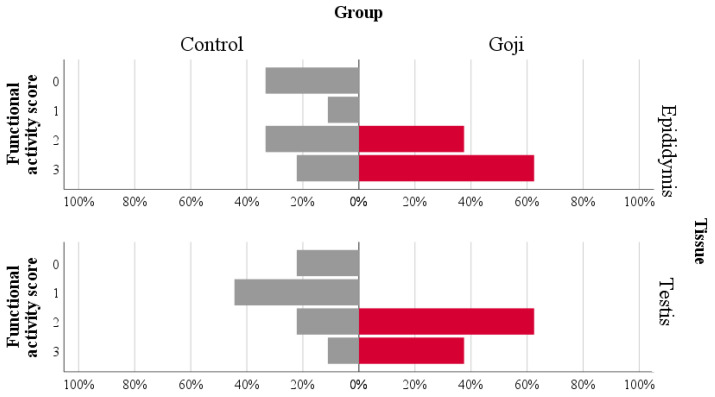
Functional activity: a population pyramid showing the relative distribution by groups (Control: gray bars on the left; Goji: red bars on the right) of functional activity scores in the epididymis (**top panel**) and testis (**bottom panel**). The eta (0.543 and 0.611 for epididymis and testis, respectively) indicated a strong association between the group and these scores.

**Figure 3:**
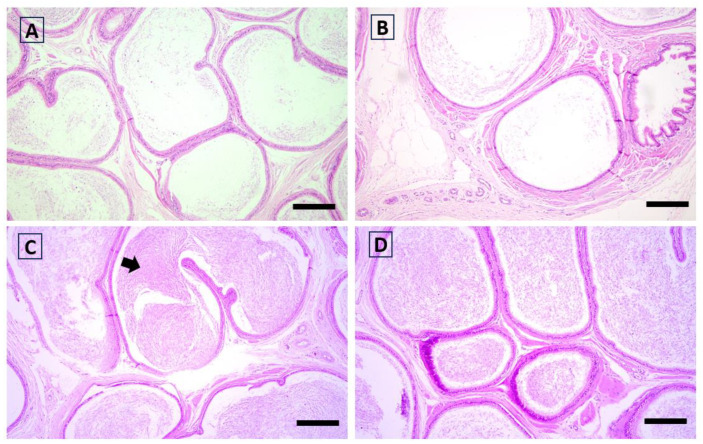
Rabbit histological aspect of the epididymis in which the difference between the Control (**A**,**B**) and Goji (**C**,**D**) groups is evident: (**A**,**B**) epididymal tubules are almost empty; (**C**,**D**) a “ball” of spermatozoa can be observed in the tubular lumen (arrow). Hematoxylin and eosin; scale bar = 150 µm.

**Figure 4:**
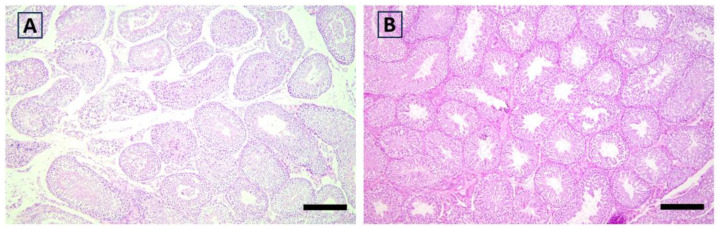
Rabbit histological aspect of the testes in the Control (**A**) and Goji (**B**) groups showing different patterns of spermatogenesis, with intense activity in the testis of (**B**). Hematoxylin and eosin; scale bar = 150 µm.

**Figure 5:**
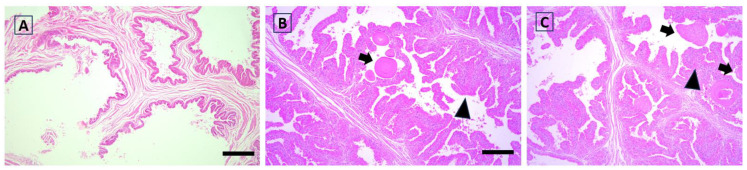
Rabbit histological aspect of the prostate in the Control (**A**) and Goji (**B**,**C**) groups showing a hyperplastic structure, with some foci of proliferating epithelium forming papillary structures with supporting stroma and extension into the glandular lumen (arrow heads). Corpora amylacea are also seen (arrows). Hematoxylin and eosin; scale bar = 150 µm.

The eta coefficient indicated a large association of functional activity scores with the group in the epididymis (eta = 0.543) and testis (eta = 0.611; Figure 2, Figure 3, Figure 4 and Figure 5). The greater proportion of the highest scores in the Goji group explained 74% and 78% of the variance in the epididymis and testis, respectively. In the testis, a medium association with the group was also found for hyperplasia (eta = 0.457), as most of the samples (78%) in the Control group had scores = 0, while a more heterogeneous distribution between the highest scores was found in the Goji group (Table S1). No other parameters were associated with nutritional treatment (Table S1).

### 3.5. Correlations between Seminal Parameters, TAC, Antioxidant Enzymes, and Interleukin-1β 

A poor (ρ < 0.3) although significant positive correlation was found between TAC and volume, GPx and concentration, GPx and rate of live spermatozoa, and IL-1β and percentage of live spermatozoa. Conversely, GPx was negatively correlated with the percentage of abnormal spermatozoa (Table 4).

Table 5 shows the correlations between seminal quality, oxidative stress, inflammatory status parameters and histological scores of the epididymis and testes. The epididymal degeneration score was positively correlated with volume and live spermatozoa, whereas those of the testis was negatively correlated with the volume. Functional activity was negatively correlated with abnormal spermatozoa both in the epididymis and testis. The functional activity of the testes also showed a positive correlation with motility and a negative one with SOD. Finally, hyperplasia of the epididymis was positively correlated with live spermatozoa, TAC, and CAT (for all *p* < 0.05). The correlations with histological scores of the other glands are shown in Table S2. Among these, the positive correlation of the functional activity score of the bulbourethral glands with motility and live spermatozoa, as well as the negative correlation of the functional activity score of the prostate with abnormal spermatozoa, can be mentioned (for all *p* < 0.05).

## 4. Discussion

This study investigated, for the first time, the effect of GB on semen quality, antioxidant and inflammatory capacity of seminal plasma, and histological structure of reproductive organs in rabbit bucks. The findings suggest that GB dietary supplementation can improve several semen traits (concentration, motile cells, live cells, and abnormal spermatozoa), although no effect on parameters related to oxidative status was found. However, the histological analyses showed noticeable effects of the GB-supplemented diet on the functional activity of the testis, as well as on the degree of hyperplasia of the epididymis and prostate, which could be linked to the better semen quality. These preliminary results suggest that GB could affect rabbit fertility and can be used for animal nutrition, although further studies are required to understand its mechanism of action and determine the optimal dose to be used.

In this pioneering work regarding male rabbits, the selection of the dose to be administered was based on previous studies conducted on female rabbits, which had shown interesting results on various aspects of reproduction and productivity [50,51,52]. The primary notable outcome of this study is thus that the 1% integration of GB did not induce any health issues in the animals. The feed proved to be palatable and was consumed by the rabbits without any problem. These findings confirm previous studies, as GB appeared to be safe at various dosages [11]. Only mild toxicity [8] and adverse effects such as urticarial-like reactions related to its consumption have been reported in humans [67]. Furthermore, the histological results demonstrated that, at the level of the genital organs of rabbits, GB not only does not cause alterations but also increases functional activity. The effects of the berry on the male reproductive tract histology under physiological conditions are little known. On the contrary, experimental models of various pathological conditions, especially in rodents, show that GB supplementation can improve the histological characteristics of the reproductive tissues, as well as the spermatic functions [36,43,44,68,69]. In the streptozotomycin-induced diabetic rats model, GB treatment ameliorated the damage in the spermatogenic tubules, increased the number of spermatogenic cells, and suppressed apoptosis in the testes [68]. *Lycium barbarum* polysaccharides ameliorated the damage in the seminiferous tubules induced by cadmium both in rats and mice [36,44]. The pretreatment with GB mitigated the degenerative changes in seminiferous tubules and improved hormonal secretion, spermatozoa concentration, and motility, as well as oxidative capacity in doxorubicin-induced testicular toxicity in rats [43]. Finally, our findings are in agreement with other studies performed on the rabbit does and fattening rabbits [50,51,61]. The findings from these studies highlight an improvement in the mitigation of the damage caused by various stressors on the reproductive system tissues; however, it remains uncertain whether this improvement is due to a direct action of the active biological substances of the berry or if it is an indirect effect, perhaps mediated by an increase in antioxidant activity within the reproductive tract, or a combination of both. 

Regardless of the mechanism of action, our findings demonstrate that parameters related to functional activity, hyperplasia, and tissue degeneration in the reproductive tract of bucks are correlated with several semen traits such as motility, number of live and abnormal spermatozoa. As a matter of fact, rabbits supplemented with GB also had better semen quality in terms of concentration, motility, vitality, and abnormal spermatozoa. Although as a trend, libido also improved. To our knowledge, the effects of GB dietary supplementation on the characteristics of semen in physiological conditions have been previously studied only by Yang et al. [58]. These authors evaluated a dried GB supplementation for 160 days in boars, finding an improvement in spermatozoa motility, concentration, and abnormality rate. On the other hand, several studies have assessed both in vivo and in vitro the impact of GB in different pathological models and various animal species, including mice, rats, and goats [59,70,71]. 

The positive effects of GB on seminal quality are often attributed to its antioxidant effect [14,18,23,72]. However, our results do not support this hypothesis as no differences between groups were found neither in the parameters indicating oxidative status nor in the cytokine. Specifically, the nutritional treatment did not affect the TAC, CAT, SOD, GPx, and IL-1β concentrations in the seminal plasma of the rabbits. Some differences between groups were found for the TAC, CAT, and SOD patterns over time, although these appear weak and inconclusive. The disagreement with previous studies may be attributed to the dose of the berry and/or the nutritional adaptation period applied in our study that may not have been sufficient to induce specific and significant effects on the oxidative status. Furthermore, species-specific characteristics and the use of the whole fruit rather than its derivatives could be influential factors. It has also been demonstrated that the GB polysaccharides administered for 5 days with dosages of 200, 400, and 600 mg/Kg improved spermatozoa mobility, concentration, percentage of normal spermatozoa morphology, and SOD concentration in mice treated with cyclophosphamide [73]. GB polysaccharides administered at a dose of 200 mg/Kg for 7 days induced an increase in antioxidant enzyme concentrations and profound protective effects against spermatogenic injury induced by bisphenol A in mice [70]. Luo et al. [55] demonstrated that GB polysaccharides, administered at a dosage of 200 mg/Kg for 15 days, enhanced sexual ability, increased antioxidant activity, and improved seminal quality in rat testes models subjected to heat-induced damage. In many studies, the improvement in semen quality of animals receiving GB was also associated with an increase in the concentration of reproductive hormones such as testosterone and LH and a reduction in the expression of genes promoting apoptosis such as BAX [40,44,55,70,74,75]. 

Goji is rich in polyphenols, flavonoids, carotenoids, and vitamin C, and it also exhibits anti-inflammatory, antisclerotic, protective, and regulatory properties, and t could exert epigenetic and gut-microbiota modulations [29,76,77]. In particular, it has been demonstrated that flavonoids, such as quercetin, improve male performance by acting on crucial mitochondrial processes of spermatozoa and enhancing the activity of the hypothalamic–pituitary–testicular axis [76,78,79]. Lycopene, a carotenoid also found in other fruits, such as ripe tomatoes and *F. sellowiana* [12,80], accumulates in seminal prostasomes and seems to improve sperm count and reduce the number of abnormal spermatozoa [80,81], playing a role in several physiological processes, mainly with protective effects (antioxidants and anti-inflammatory) [80,82]. Moreover, beyond the specific effects of GB, the enhancement in reproductive functions could reflect the overall improved health associated with the consumption of these substances [29]. It could be speculated that GB supplementation may influence the secretion of reproductive hormones and/or their receptors, consequently, impacting the histological conditions of the male reproductive tract tissues and improving the spermatozoa concentration and mobility, apoptosis rate, and number of abnormal cells. Hormonal changes could also explain the improved libido in the Goji group. In the present study, an evaluation of the concentration of reproductive hormones was not conducted, although the histological results clearly demonstrate that the diet supplemented with GB improves the histological characteristics of the tissues of the reproductive organs and associated glands.

Thus, the main limitation of the study is the lack of evaluation of hormones, receptors, enzymes, and genes directly involved in reproductive functions. Moreover, a higher supplementation dosage of the berry and/or a longer nutritional adaptation period can be employed to evaluate the antioxidant and anti-inflammatory effect of GB and to understand any alternative mechanisms of action on the reproductive system of rabbit bucks. Finally, we recognize that the incorporation of goji in animal feed may be intended for a niche market; however, a cost–benefit analysis should still be carried out.

## 5. Conclusions

The dietary administration of GB improved several semen traits, including spermatozoa motility and concentration, as well as live and abnormal cells. Histological changes were also observed, including increased functional activity in the testis, hyperplasia of the epididymis and prostatic epithelium (with increased intra-acinar content of *corpora amylacea*), along with a higher concentration of spermatozoa within dilated epididymal tubules. Conversely, its effect on the antioxidative and inflammatory capacity of seminal plasma has not been demonstrated. Further studies may involve a higher percentage of GB diet integration and a longer nutritional adaptation period as the antioxidant effect could be dose and time dependent. 

The use of this natural and nutraceutical product is safe and does not have particular side effects both at the systemic and local levels. For this reason, GB could potentially be a new ingredient for the rabbit feed. Dietary supplementation with the berry could be an innovative strategy to increase the fertility and welfare of animals, as well as the profitability of farmers, although a cost analysis should be performed. Further studies are in progress to understand GB’s mechanism of action including its effects on the antioxidant and inflammatory capacity of reproductive tract tissues and reproductive hormones.

## Figures and Tables

**Table 1:** Formulation and chemical composition (as fed) of control and experimental diet supplemented with 1% GB.

Ingredients/Analytical Data	Diet	
	Control	Goji
**Ingredients ^1^**		
Wheat bran	30.0	29.5
Dehydrated alfalfa meal	42.0	41.5
Barley	9.6	9.6
Sunflower meal	4.6	4.6
Rice bran	4.0	4.0
Soybean meal	4.0	4.0
Calcium carbonate	2.0	2.0
Cane molasses	2.0	2.0
Dicalcium phosphate	0.7	0.7
Vitamin–mineral premix ^2^	0.4	0.4
Soybean oil	0.4	0.4
Salt	0.3	0.3
Goji berries	-	1
**Analytical data ^1^**		
Crude protein	15.74	15.64
Ether extract	2.25	2.23
Ash	9.28	9.36
Starch	16.86	17.07
NDF	38.05	38.55
ADF	19.54	19.60
ADL	4.01	4.31
**Digestible Energy ^3^**	2464	2463

^1^ As a percentage (%). ^2^ Per kg diet: vitamin A 11,000 IU; vitamin D3 2000 IU; vitamin B1 2.5 mg; vitamin B2 4 mg; vitamin B6 1.25 mg; vitamin B12 0.01 mg; alpha-tocopherol acetate 50 mg; biotin 0.06 mg; vitamin K 2.5 mg; niacin 15 mg; folic acid 0.30 mg; D-pantothenic acid 10 mg; choline 600 mg; Mn 60 mg; Fe 50 mg; Zn 15 mg; I 0.5 mg; Co 0.5 mg. ^3^ As Kcal/kg. Estimated by Maertens et al. [62].

**Table 2:** Descriptive statistics and generalized linear model results for the seminal parameters. The values are the means and standard error (SE) at each time point, as well as the marginal means according to the group.

Parameter	Time	Group		*p*-Value		
		Control	Goji	Group	Time	Group × Time
Volume (mL)	T1	0.95 ± 0.19	0.97 ± 0.07	0.791	0.733	0.413
	T2	0.98 ± 0.12	0.80 ± 0.12			
	T3	0.83 ± 0.10	1.01 ± 0.09			
	T4	0.98 ± 0.08	1.03 ± 0.17			
	T5	0.96 ± 0.16	1.03 ± 0.06			
	Marginal means ± SE	0.94 ± 0.06	0.97 ± 0.05			
Concentration (spermatozoa/mL; 10^6^)	T1	189 ± 36	263 ± 36	**0.040**	0.140	0.339
	T2	176 ± 33	309 ± 49			
	T3	253 ± 45	302 ± 23			
	T4	249 ± 47	310 ± 33			
	T5	268 ± 50	354 ± 45			
	Marginal means ± SE	227 ^a^ ± 19	308 ^b^ ± 17			
Motility (%)	T1	81 ± 6	95 ± 1	**0.001**	0.081	0.096
	T2	87 ± 4	94 ± 1			
	T3	88 ± 3	94 ± 1			
	T4	87 ± 3	92 ± 3			
	T5	93 ± 1	94 ± 2			
	Marginal means ± SE	87 ^a^ ± 2	94 ^b^ ± 1			
Live spermatozoa (%)	T1	91.1 ± 4.0	97.0 ± 0.6	**0.010**	0.283	**0.003**
	T2	95.0 ± 1.5	92.4 ± 2.8			
	T3	93.2 ± 1.8	95.1 ± 1.1			
	T4	95.2 ± 1.0	97.6 ± 0.5			
	T5	93.1 ± 1.3	94.1 ± 2.0			
	Marginal means ± SE	93.5 ^a^ ± 1.0	95.2 ^b^ ± 0.8			
Abnormal spermatozoa (%)	T1	7.6 ± 1.2	5.3 ± 1.1	**<0.001**	**<0.001**	0.277
	T2	7.9 ± 0.9	6.6 ± 1.1			
	T3	8.6 ± 2.2	4.6 ± 1.5			
	T4	19.2 ± 2.1	11.2 ± 1.5			
	T5	13.2 ± 0.8	9.9 ± 1.3			
	Marginal means ± SE	11.3 ^b^ ± 0.9	7.5 ^a^ ± 0.7			
Reaction time (s) *	T1	11.11 ± 2.89	6.52 ± 1.74	0.066	**<0.001**	0.340
	T2	4.50 ± 1.33	4.27 ± 2.81			
	T3	5.59 ± 1.88	1.88 ± 0.44			
	T4	2.99 ± 1.75	1.89 ± 0.66			
	T5	3.23 ± 0.99	0.99 ± 0.23			
	Marginal means ± SE	5.48 ± 0.92	3.02 ± 0.77			

For each row, the values followed by the same letter do not have statistically significant differences (at the level of 0.05). If no letters are present, the differences were not statistically significant. *p*-Values in bold denote statistical significance at the level of 0.05. * The interval from the introduction of the “teaser” doe into the male’s cage to the first ejaculation was considered an indication of libido [63].

**Table 3:** Antioxidant and inflammatory parameters were evaluated in the seminal plasma of rabbits at different sampling times (values are given as the means ± SE).

Parameter	Time	Group		*p*-Value		
		Control	Goji	Group	Time	Group × Time
TAC	T1	4.32 ± 0.74	5.78 ± 1.00	0.808	**<0.001**	**<0.001**
	T2	4.79 ± 0.80	3.71 ± 0.59			
	T3	4.51 ± 0.45	6.38 ± 0.45			
	T4	5.88 ± 0.21	4.52 ± 0.32			
	T5	7.42 ± 0.67	7.17 ± 0.46			
	Marginal means ± SE	5.38 ± 0.31	5.51 ± 0.33			
CAT	T1	161.58 ± 36.27	294.60 ± 82.54	0.296	**0.004**	**<0.001**
	T2	109.96 ± 19.63	169.99 ± 37.63			
	T3	151.16 ± 27.85	190.70 ± 23.51			
	T4	150.00 ± 17.72	130.47 ± 33.58			
	T5	190.94 ± 39.92	171.74 ± 34.39			
	Marginal means ± SE	152.73 ± 13.22	192.59 ± 22.40			
SOD	T1	150.42 ± 19.61	173.36 ± 15.26	0.115	0.261	**0.008**
	T2	169.52 ± 19.06	165.70 ± 28.08			
	T3	194.43 ± 27.69	161.23 ± 20.48			
	T4	195.05 ± 16.33	161.41 ± 11.13			
	T5	212.45 ± 31.38	158.22 ± 15.65			
	Marginal means ± SE	184.38 ± 10.55	163.98 ± 8.10			
GPx (U/mL)	T1	47.54 ± 24.85	182.42 ± 54.69	0.084	0.565	0.094
	T2	44.15 ± 12.07	86.23 ± 21.33			
	T3	137.53 ± 44.84	210.12 ± 98.47			
	T4	51.79 ± 19.01	135.62 ± 35.89			
	T5	75.56 ± 16.67	85.00 ± 38.92			
	Marginal means ± SE	71.31 ± 12.32	141.26 ± 25.78			
IL-1β (pg/mL)	T1	1698.47 ± 200.79	1901.66 ± 199.64	0.630	0.561	0.205
	T2	1594.85 ± 264.49	1834.93 ± 250.40			
	T3	1742.88 ± 274.36	1887.11 ± 229.48			
	T4	1998.12 ± 184.93	1740.87 ± 208.84			
	T5	1833.63 ± 183.01	1945.94 ± 150.24			
	Marginal means ± SE	1775.30 ± 98.66	1862.10 ± 89.86			

TAC: total antioxidant capacity; CAT: catalase; GPx: glutathione peroxidase; SOD: superoxide dismutase; IL-1 β: interleukin-1 β. CAT, GPx, and IL-1β were analyzed after root square transformation. Letters indicating differences between the groups are not present because pairwise comparisons were not significant. *p*-Values in bold denote statistical significance at the level of 0.05.

**Table 4:** Spearman’s rho coefficient for the correlations between seminal parameters, total antioxidant capacity, antioxidant enzymes, and interleukin-1β.

	Concentration	Motility	Live Spermatozoa	Abnormal Spermatozoa	Reaction Time	TAC	CAT	SOD	GPx	IL-1β
Volume	0.182	0.131	0.233 *	−0.118	0.025	0.219 *	−0.074	0.031	0.070	0.091
Concentration		0.572 **	0.236 *	−0.195	−0.256 *	−0.025	0.045	−0.008	0.233 *	0.213
Motility			0.348 **	−0.214 *	0.027	0.015	0.065	−0.057	0.171	0.086
Live spermatozoa				−0.382 **	0.020	−0.014	0.118	0.020	0.237 *	0.288 **
Abnormal spermatozoa					−0.214	0.077	−0.108	−0.056	−0.279 *	−0.143
Reaction time						−0.082	−0.011	−0.171	−0.205	−0.178
TAC							0.422 **	0.150	0.121	0.133
CAT								0.262 *	0.365 **	0.131
SOD									0.346 **	0.162
GPx										0.265 *

TAC: total antioxidant capacity; CAT: catalase; GPx: glutathione peroxidase; SOD: superoxide dismutase; IL-1β: interleukin-1β. The values in bold denote the statistical significance of the correlation (2-tailed): ** At the 0.01 level; * At the 0.05 level.

**Table 5:** Spearman’s rho coefficient for the correlations between histological scores evaluated on epididymis and testis and seminal parameters, total antioxidant capacity, antioxidant enzymes, and interleukin-1β.

Tissue	Parameter	Volume	Concentration	Motility	Live Spermatozoa	Abnormal Spermatozoa	Reaction Time	TAC	CAT	SOD	GPx	IL-1β
**Epididymis**	**Degeneration**	0.26	0.489 *	0.324	0.568 *	−0.251	−0.293	0.216	0.399	−0.175	0.192	0.011
	**Functional activity**	−0.133	0.229	0.453	0.164	−0.507 *	−0.13	0.12	0.284	−0.248	0.395	0.113
	**Hyperplasia**	0.222	0.214	0.443	0.639 **	−0.313	−0.138	0.542 *	0.535 *	−0.374	0.374	0.351
**Testis**	**Degeneration**	−0.606 **	−0.046	−0.334	−0.164	0.299	−0.12	−0.253	−0.017	0.148	−0.24	−0.413
	**Functional activity**	−0.037	0.242	0.595 *	0.273	−0.501 *	−0.277	0.088	0.327	−0.493 *	0.452	−0.121
	**Hyperplasia**	−0.277	0.108	0.150	0.276	−0.389	−0.055	0.284	0.482	−0.161	0.223	0.169

TAC: total antioxidant capacity; CAT: catalase; GPx: glutathione peroxidase; SOD: superoxide dismutase; IL-1β: interleukin-1β. The values in bold denote the statistical significance of the correlation (2-tailed): ** at the 0.01 level; * at the 0.05 level.

## Data Availability

The data presented in this study are available on request from the corresponding author.

## Supplementary Materials

The following supporting information can be downloaded at: https://www.mdpi.com/article/10.3390/antiox12111959/s1, Figure S1: Evaluation of the color of the semen samples. The values are the percentages of the samples for each time point; Table S1: Crosstabulation of the histological scores by group according to the tissue and parameter; Table S2: Spearman’s rho coefficient for the correlations between histological scores evaluated in urethral bulb glands, prostate and seminal vesicles, and seminal parameters, total antioxidant capacity, antioxidant enzymes, and interleukin-1β.
