# Supplementary material for: Goji Berry in the Diet of the Rabbit Buck: Effects on Semen Quality, Oxidative Status and Histological Features of the Reproductive Tract
Source: Antioxidants (Basel). 2023 Nov 2;12(11):1959. doi: 10.3390/antiox12111959 (PMC10669443; doi:10.3390/antiox12111959)

## Supplementary material

**Table S1:** Crosstabulation of the histological scores by group according to the tissue and parameter.

| Tissue | Parameter | Score | Group Control Count | Group Control Column N % | Group Goji Count | Group Goji Column N % | P value* | Eta |
|---|---|---|---|---|---|---|---|---|
| Epididymis | **Phlogosis** | **0** | 9 | 100.0% | 8 | 100.0% | - | - |
| | **Degeneration** | **0** | 8 | 88.9% | 6 | 75.0% | 0.718 | 0.241 |
| | | **1** | 1 | 11.1% | 1 | 12.5% | | |
| | | **2** | 0 | 0.0% | 1 | 12.5% | | |
| | **Functional activity** | **0** | 3 | 33.3% | 0 | 0.0% | 0.194 | **0.543 (eta$^2$=0.737)** |
| | | **1** | 1 | 11.1% | 0 | 0.0% | | |
| | | **2** | 3 | 33.3% | 3 | 37.5% | | |
| | | **3** | 2 | 22.2% | 5 | 62.5% | | |
| | **Hyperplasia** | **0** | 7 | 77.8% | 5 | 62.5% | 0.462 | 0.291 |
| | | **1** | 2 | 22.2% | 1 | 12.5% | | |
| | | **2** | 0 | 0.0% | 2 | 25.0% | | |
| | **Necrosis** | **0** | 9 | 100.0% | 8 | 100.0% | - | - |
| **Urethral bulb glands** | **Phlogosis** | **0** | 8 | 88.9% | 7 | 87.5% | 1.000 | 0.095 |
| | | **1** | 0 | 0.0% | 1 | 12.5% | | |
| | | **2** | 1 | 11.1% | 0 | 0.0% | | |
| | **Degeneration** | **0** | 6 | 66.7% | 7 | 87.5% | 1.000 | 0.280 |
| | | **1** | 2 | 22.2% | 1 | 12.5% | | |
| | | **2** | 1 | 11.1% | 0 | 0.0% | | |
| | **Functional activity** | **0** | 1 | 11.1% | 2 | 25.0% | 0.741 | 0.023 |
| | | **1** | 5 | 55.6% | 2 | 25.0% | | |
| | | **2** | 2 | 22.2% | 3 | 37.5% | | |
| | | **3** | 1 | 11.1% | 1 | 12.5% | | |
| | **Hyperplasia** | **0** | 3 | 33.3% | 4 | 50.0% | 0.741 | 0.000 |
| | | **1** | 3 | 33.3% | 1 | 12.5% | | |
| | | **2** | 3 | 33.3% | 2 | 25.0% | | |
| | | **3** | 0 | 0.0% | 1 | 12.5% | | |
| | **Necrosis** | **0** | 9 | 100.0% | 8 | 100.0% | - | - |

[continues]

| | | | | | | | | |
|---|---|---|---|---|---|---|---|---|
| **Prostate** | **Phlogosis** | **0** | 9 | 100.0% | 8 | 100.0% | - | - |
| | **Degeneration** | **0** | 8 | 88.9% | 6 | 75.0% | 0.576 | 0.182 |
| | | **1** | 1 | 11.1% | 2 | 25.0% | | |
| | **Functional activity** | **0** | 2 | 22.2% | 0 | 0.0% | 0.793 | 0.238 |
| | | **1** | 4 | 44.4% | 4 | 50.0% | | |
| | | **2** | 2 | 22.2% | 3 | 37.5% | | |
| | | **3** | 1 | 11.1% | 1 | 12.5% | | |
| | **Hyperplasia** | **0** | 3 | 33.3% | 2 | 25.0% | 1.000 | 0.181 |
| | | **1** | 3 | 33.3% | 2 | 25.0% | | |
| | | **2** | 2 | 22.2% | 2 | 25.0% | | |
| | | **3** | 1 | 11.1% | 2 | 25.0% | | |
| | **Necrosis** | **0** | 9 | 100.0% | 8 | 100.0% | - | - |
| **Testis** | **Phlogosis** | **0** | 9 | 100.0% | 8 | 100.0% | - | - |
| | **Degeneration** | **0** | 7 | 77.8% | 7 | 87.5% | 1.000 | 0.191 |
| | | **1** | 1 | 11.1% | 1 | 12.5% | | |
| | | **2** | 1 | 11.1% | 0 | 0.0% | | |
| | **Functional activity** | **0** | $2_{a}$ | 22.2% | $0_{a}$ | 0.0% | 0.046 | **0.611 (eta$^2$=0.782)** |
| | | **1** | $4_{a}$ | 44.4% | $0_{b}$ | 0.0% | | |
| | | **2** | $2_{a}$ | 22.2% | $5_{a}$ | 62.5% | | |
| | | **3** | $1_{a}$ | 11.1% | $3_{a}$ | 37.5% | | |
| | **Hyperplasia** | **0** | 7 | 77.8% | 3 | 37.5% | 0.302 | **0.457** |
| | | **1** | 1 | 11.1% | 1 | 12.5% | | |
| | | **2** | 1 | 11.1% | 3 | 37.5% | | |
| | | **3** | 0 | 0.0% | 1 | 12.5% | | |
| | **Necrosis** | **0** | 9 | 100.0% | 8 | 100.0% | - | - |
| **Seminal vesicles** | **Phlogosis** | **0** | 9 | 100.0% | 7 | 87.5% | 0.471 | 0.265 |
| | | **2** | 0 | 0.0% | 1 | 12.5% | | |
| | **Degeneration** | **0** | 8 | 88.9% | 7 | 87.5% | 1.000 | 0.022 |
| | | **1** | 1 | 11.1% | 1 | 12.5% | | |
| | **Functional activity** | **0** | 4 | 44.4% | 2 | 25.0% | 0.445 | 0.116 |
| | | **1** | 2 | 22.2% | 4 | 50.0% | | |
| | | **2** | 2 | 22.2% | 0 | 0.0% | | |
| | | **3** | 1 | 11.1% | 2 | 25.0% | | |
| | **Hyperplasia** | **0** | 5 | 55.6% | 3 | 37.5% | 0.332 | 0.388 |
| | | **1** | 4 | 44.4% | 2 | 25.0% | | |
| | | **2** | 0 | 0.0% | 2 | 25.0% | | |
| | | **3** | 0 | 0.0% | 1 | 12.5% | | |
| | **Necrosis** | **0** | 9 | 100.0% | 8 | 100.0% | - | - |

* p value from Fisher’s exact
- No computed as variable is constant
Values followed by the same subscript letter in each row do not differ ($p \leq 0.05$; z-test).
Eta values indicating medium or large association are in bold.

**Table S2:** Spearman's rho coefficient for the correlations between histological scores evaluated in urethral bulb glands, prostate and seminal vesicles, and seminal parameters, total antioxidant ca-pacity, antioxidant enzymes, and interleukin-1β.

| Tissue | Parameter | Volume | Concentration | Motility | Live cells | Abnormal cells | Reaction time | TAC | CAT | SOD | GPx | IL1beta |
|---|---|---|---|---|---|---|---|---|---|---|---|---|
| Urethral bulb glands | Degeneration | **0.583*** | -0.119 | -0.132 | 0.079 | 0 | 0.212 | 0.364 | 0.404 | 0.132 | 0.02 | -0.304 |
| | Functional activity | 0.384 | 0.47 | **0.589*** | **0.599*** | -0.385 | 0.068 | 0.09 | -0.096 | **-0.563*** | -0.013 | -0.132 |
| | Hyperplasia | 0.296 | 0.058 | -0.08 | 0.057 | 0.032 | 0.236 | -0.049 | 0.07 | -0.056 | **-0.518*** | **-0.570*** |
| Prostate | Degeneration | -0.095 | 0.315 | 0.079 | 0.300 | -0.315 | -0.189 | 0.378 | **0.567*** | 0.157 | 0.441 | -0.031 |
| | Functional activity | 0.053 | 0.301 | 0.385 | 0.464 | **-0.504*** | -0.109 | 0.477 | **0.570*** | -0.100 | **0.531*** | 0.19 |
| | Hyperplasia | 0.127 | 0.285 | 0.152 | 0.267 | -0.227 | 0.242 | 0.372 | **0.571*** | 0.161 | 0.013 | -0.061 |
| Seminal vesicles | Degeneration | -0.112 | 0.112 | 0.056 | 0.037 | -0.373 | 0.335 | 0.298 | 0.335 | 0.037 | 0.149 | -0.075 |
| | Functional activity | 0.02 | 0.199 | 0.261 | 0.229 | -0.194 | -0.086 | 0.311 | 0.376 | 0.134 | 0.175 | 0.023 |
| | Hyperplasia | -0.159 | 0.354 | 0.270 | 0.236 | **-0.529*** | -0.056 | 0.340 | 0.431 | 0.038 | 0.104 | -0.230 |

TAC: Total Antioxidant Capacity; CAT: Catalase; GPx: Glutathione Peroxidase; SOD: Superoxidase Dismutase; IL-1β: Interleukin-1 β. Values in bold denote statistical significance of the correlation (2-tailed): * at the 0.05 level.

**Figure S1:** Evaluation of the color of the semen samples. The values are the percentages of the samples for each time point.

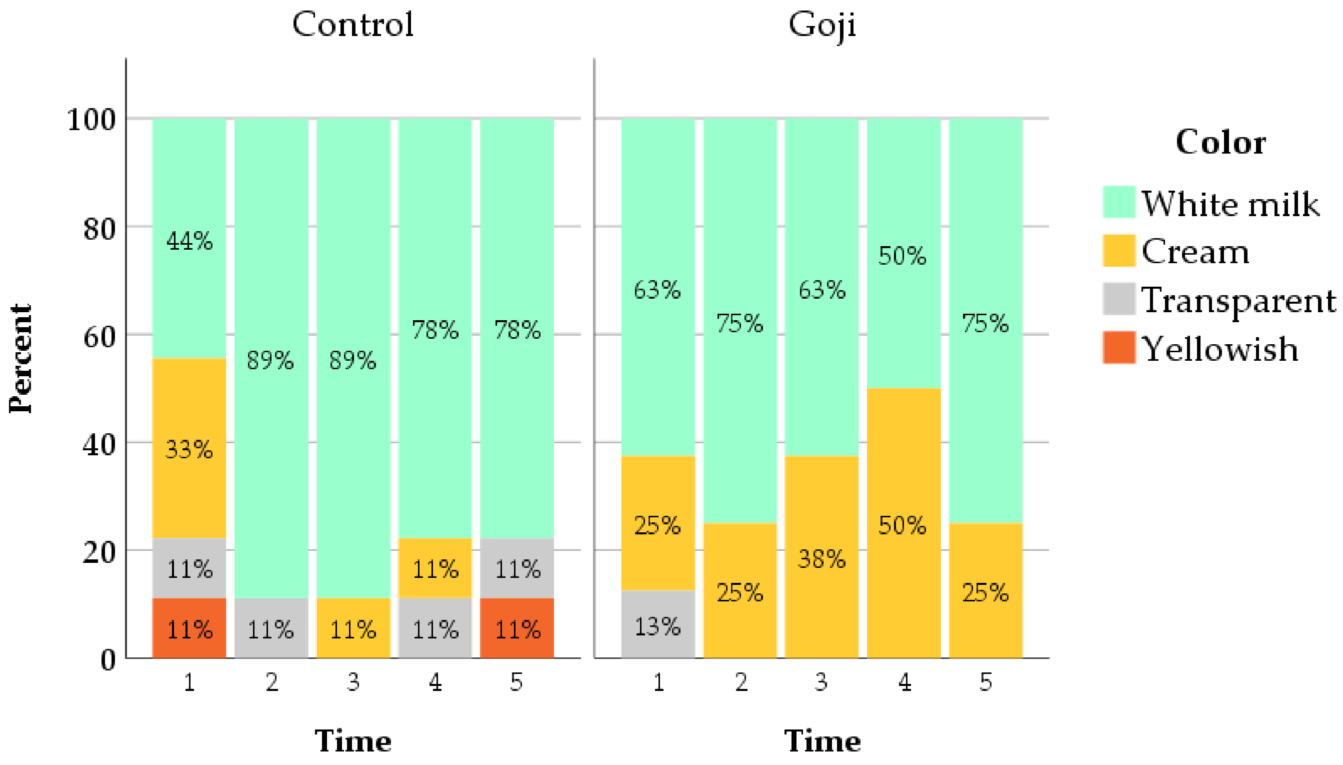

Supplement: Supplementary file 1 [file antioxidants-12-01959-s001.zip › antioxidants-2664913-supplementary.pdf]
